# Reverse regulation of soluble receptor for advanced glycation end products and proinflammatory factor resistin and S100A12 in Kawasaki disease

**DOI:** 10.1186/ar4094

**Published:** 2012-11-21

**Authors:** Yanqi Qi, Fangqi Gong, Qing Zhang, Chunhong Xie, Wei Wang, Songling Fu

**Affiliations:** 1 Children's Hospital, Zhejiang University School of Medicine, Hangzhou, Zhejiang, 310003 China

## Abstract

**Introduction:**

Kawasaki disease (KD), an acute febrile disease, characterized by systemic vasculitis, predominantly affects infants and children under 5 years of age. Coronary artery lesions (CALs) are its most critical complication, and the etiology remains unknown yet. In order to explore the value of resistin, S100A12 and soluble receptor for advanced glycation end products (sRAGE) in the pathophysiology of KD, we studied the serum levels of resistin, S100A12 and sRAGE in different stages of KD.

**Methods:**

Serum levels of resistin, S100A12 and sRAGE were measured by enzyme-linked immunosorbent assay (ELISA) method in 15 healthy children and 40 KD patients at acute, afebrile and subacute stage.

**Results:**

The resistin and S100A12 levels, including the ratio of resistin to sRAGE and S100A12 to sRAGE increased significantly in the acute stage, and decreased progressively in the afebrile and subacute stage. However, the sRAGE levels decreased significantly in the acute stage, and increased progressively in the afebrile and subacute stage. In the acute, afebrile and subacute stage, the resistin levels were higher in intravenous immunoglobulin (IVIG) non-responders (0.64 ± 0.30, 0.48 ± 0.35, 0.28 ± 0.19, × 10^2 ^ng/ml) than in IVIG responders (0.35 ± 0.24, 0.21 ± 0.19, 0.12 ± 0.05, × 10^2 ^ng/ml). In the acute and subacute stage, the S100A12 levels were higher in IVIG non-responders (7.92 ± 2.61, 4.98 ± 4.75, × 10^2 ^ng/ml) than in IVIG responders (5.05 ± 3.22, 2.35 ± 2.26, × 10^2 ^ng/ml). In the afebrile and subacute stage, the sRAGE levels were lower in IVIG non-responders (3.51 ± 2.64, 3.65 ± 3.27, × 10^2 ^pg/ml) than in IVIG responders (6.00 ± 2.78, 7.19 ± 2.88, × 10^2 ^pg/ml). The resistin levels were positively correlated with S100A12 levels. The sRAGE levels were negatively related with S100A12 and resistin levels.

**Conclusions:**

Resistin, S100A12 and sRAGE are involved in the pathophysiology of KD.

## Introduction

Kawasaki disease (KD), an acute febrile disease characterized by systemic vasculitis, predominantly affects infants and children under 5 years of age [1]. Coronary artery lesions (CALs) are its most critical complication [2], and as yet the etiology remains unknown.

Human resistin is a 12.5 kDa cysteine-rich peptide of bioactive molecules produced by the adipose tissue, monocytes and macrophages [3,4]. Although considered an adipokine, importantly, resistin is expressed in macrophages and plays important roles in systemic inflammation; it also appears to be a predominant pro-inflammatory protein associated with both acute and chronic inflammation [5-9]. Resistin may have a potential role in the development of endothelial dysfunction [10], thrombosis, angiogenesis, inflammation and smooth muscle cell dysfunction in cardiovascular disease (CVD) and atherosclerosis [11-13]. There is increasing evidence from human clinical and experimental studies that resistin has a pathogenic role in the development and progression of atherosclerosis, coronary artery disease (CAD), and even heart failure [14]. Resistin may have a pivotal role in the pathophysiology of cardiovascular events.

S100A12 is a member of the S100 family of calcium-binding proteins. It is a key ligand of the receptor for advanced glycation end products (RAGE), which is currently secreted by neutrophils, with low expression on lymphocytes and monocytes [15]. S100A12 is also called EN-RAGE (extracellular newly identified RAGE-binding protein), proposed to stress its role as a receptor-mediated signaling pathway. S100A12 binding with RAGE can activate the NF-κB pathway to produce endothelial damage and promote KD [16]. S100A12 is over-expressed at local inflammatory sites, and its serum concentrations are associated with individual disease activity [17].

It has been identified that soluble RAGE (sRAGE) corresponds to the extracellular domain of circulating RAGE in humans [18]. sRAGE has the same ligand-binding specificity as RAGE and may serve as a competitor by binding pro-inflammatory ligands, and consequently, preventing them from reaching membrane RAGE even preventing the development of inflammatory disease.

Nevertheless, the significance of these critical inflammation proteins to the risk of CALs or intravenous immunoglobulin (IVIG)-resistance in KD remains largely unknown. We found that S100A12 and RAGE expression on circulating endothelial cell surfaces increased significantly in KD [19]. In order to explore the value of resistin, S100A12 and protector sRAGE in the pathophysiology of KD, we studied the serum levels of resistin, S100A12 and sRAGE in different stages of KD.

## Materials and methods

The study was approved by the ethical committee of the Children's Hospital, Zhejiang University School of Medicine, China and was based on the institution's guidelines for human studies. The investigation conformed to the principles outlined in the Declaration of Helsinki. Informed consent was obtained from the patients' parents.

### Subjects

Forty patients with KD treated at the Children's Hospital, Zhejiang University School of Medicine were included in this study. All patients fulfilled the diagnostic criteria [20] and were treated with IVIG at 1 g/kg/d for 2 days and oral aspirin at 30 to 50 mg/kg/d. After 3 to 5 days of treatment when the patient's temperature returned to normal, the dose of aspirin was reduced to 3 to 5 mg/kg/d for 12 weeks. There were four IVIG-resistant KD patients, whose temperature was still higher than 38°C after 48 hours of standard treatment, so they were continued on IVIG at 1 g/kg/d for another 2 days. Nine patients, including five girls and four boys, had CALs (defined as coronary artery z-score > 5) [21] identified by echocardiography during the disease process. Six patients had CALs in both the left and right coronary arteries, two in the left coronary artery, and one in the right coronary artery. The CALs in the majority of KD patients were temporary and disappeared within 3 months, while in two patients they persisted more than 3 months. Dipyridamole and warfarin were given to patients with CALs depending on the severity of the lesions.

The disease process between 4 to 9 days, 10 to 13 days and 14 to 21 days was defined as acute stage (at the time of diagnosis on admission, prior to the initiation of IVIG treatment), afebrile stage (after IVIG application and the patients' temperature had returned to normal for more than 3 days), and subacute stage (patients had been discharged from hospital). Fifteen healthy children attending for a routine health examination were enrolled into the control group.

### Laboratory analysis

Blood samples were respectively collected in acute, afebrile and subacute stages of patients and in healthy children. Two milliliters of blood was collected from the ulnar vein. The samples were centrifuged to yield serum immediately, and were then kept at -20°C until analysis. White blood cell (WBC) counts, platelet counts, serum C-reactive protein (CRP) and erythrocyte sedimentation rate (ESR) were measured by conventional methods in our hospital laboratory. Serum levels of resistin, S100A12, and sRAGE were measured by ELISA according to the manufacturer's instructions. Briefly, antibody-antigen reactions were used to capture special proteins. Measurements were performed in duplicate and the results were averaged.

We used the following reagents: human resistin immunoassay (catalog number DRSN00, Quantikine, R&D Systems, Minneapolis, MN, USA); S100A12/EN-RAGE ELISA Kit (catalog number CY-8058, CircuLex™, Nagano, Japan); ELISA kit for measuring sRAGE (catalog number DRG00, Quantikine, R&D Systems, Minneapolis, MN, USA).

### Statistical analysis

SPSS 16.0 software package was used for all statistical analyses. Except where indicated otherwise, the results were expressed as mean (SD). Normal data analysis was performed with analysis of variance (ANOVA) followed by least significant difference (LSD) test for multiple comparisons; nonparametric data were analyzed by the Mann-Whitney test. Correlations were analyzed using the partial correlation test with age and gender as control variables. Differences in parameters were considered significant when the *P*-values were ≤ 0.05.

## Results

### Characteristics of the KD patients and healthy children

The clinical characteristics of 40 KD patients and 15 healthy children are summarized in Table 1. Resistin, the product of resistin and S100A12, the ratio of resistin to sRAGE, and the ratio of S100A12 to sRAGE increased significantly in the acute stage of KD, and decreased with the course of the disease; there were no significant differences between patients with KD in the afebrile and subacute stages compared with the control group. S100A12 increased significantly in the acute and afebrile stage of KD, reaching the peak in the acute stage before IVIG treatment, and decreased with the course of the disease; there were no significant differences between patients in the subacute stage of KD compared with the control group. sRAGE levels decreased significantly in the acute, afebrile and subacute stage of KD. Although the sRAGE levels increased progressively with the course of the disease, the sRAGE level in the subacute stage remained lower than in the control group.

**Table 1:** Characteristics of the patients with Kawasaki disease (KD) and healthy children (control group)

Characteristic	Kawasaki disease						Control
	Acute stage	*P*	Afebrile stage	*P*	Subacute stage	*P*	
Boys/girls, number	22/18					NS	11/4
Age, months (range)	23.2 (5.1-72.0)					NS	63.7 (2.6-109.0)
Days of fever (range)	6.3 (3.0-15.0)					--	--
Resistin, 10^2 ^ng/ml	0.38 (0.25)	0.001	0.24 (0.22) **	0.360	0.14 (0.09) **^▲^	0.405	0.19 (0.16)
S100A12, 10^2 ^ng/ml	5.33 (3.23)	0.001	4.07 (3.52) *	0.031	2.62 (2.64) **^▲^	0.542	2.05 (1.81)
sRAGE, 10^2 ^pg/ml	3. 00 (1.91)	0.000	5.75 (2.83) **	0.001	6.83 (3.07) **	0.018	9.04 (5.28)
Resistin × S100A12, 10^4^	2.61 (2.58)	0.000	1.20 (1.19) **	0.122	0.24 (0.23) **^▲^	0.877	0.32 (0.26)
Resistin/sRAGE, 10^2^	2.13 (1.89)	0.000	0.39 (0.28) **	0.708	0.21 (0.14) **	0.884	0.26 (0.21)
S100A12/sRAGE, 10^3^	2.61 (2.02)	0.000	0.84 (0.69) **	0.107	0.30 (0.25) **	0.835	0.23 (0.19)
WBCs, 10^4^/ul	1.39 (0.49)	--	1.01 (0.51) **	--	1.01 (0.51) **	--	--
Neutrophils, %	55.6 (18.8)	--	33.2 (17.2) **	--	33.4 (17.6) **	--	--
Lymphocytes, %	32.5 (15.1)	--	50.9 (16.6) **	--	54.3 (16.7) **	--	--
Monocytes, %	7.81 (3.98)	--	11.6 (16.6)	--	8.77 (2.94)	--	--
Platelets, 10^5^/ul	4.23 (1.44)	--	5.68 (2.09) **	--	6.79 (1.87) ^**▲▲^	--	--
CRP, mg/l	75.1 (49.1)	--	25.0 (33.8) **	--	3.25 (4.12) ^**▲▲^	--	--
ESR, mm/h	67.7 (29.7)	--	78.8 (30.4) **	--	59.2 (28.3) ^▲▲^	--	--

Results are mean (SD) unless stated otherwise. All *P*-values are for patients versus healthy controls. NS, not significant; sRAGE, soluble receptor for advanced glycation end products; WBCs, white blood cells; CRP, C-reactive protein; ESR, erythrocyte sedimentation rate. *P < 0.05, **P < 0.01 compared with acute stage; ^▲^*P *< 0.05, ^▲▲^*P *< 0.01 compared with afebrile stage.

### Serum levels of resistin, S100A12, sRAGE in IVIG responder and non-responder KD patients

**Figure 1:**
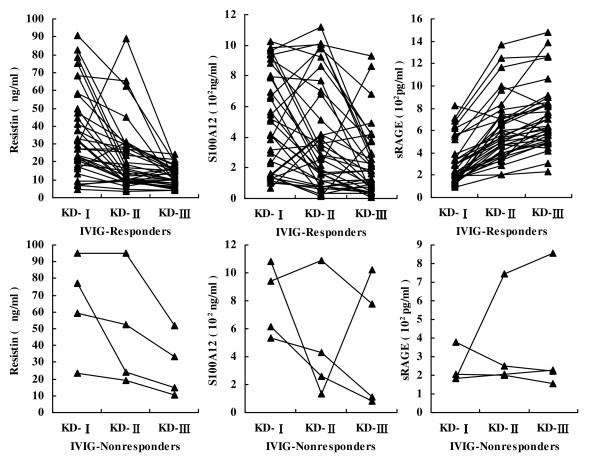
**Resistin, S100A12 and soluble receptor for advanced glycation end products (sRAGE) levels at different stages of Kawasaki disease (KD) in patients treated with intravenous immunoglobulin (IVIG)**. KD-I, acute stage; KD-II, afebrile stage; KD-III, subacute stage.

Serum levels of resistin, S100A12, and sRAGE in IVIG responder and non-responder KD patients are shown in Table 2 and Figure 1. Not all KD patients were sensitive to the treatment of IVIG. There were four IVIG-resistant KD patients (one boy and three girls). In the acute, afebrile and subacute stages of KD, although the resistin levels decreased progressively with the course of disease, they were higher in IVIG non-responders than in IVIG responders. The S100A12 levels also decreased progressively with the course of disease, but in the acute and subacute stages of KD, the S100A12 levels were higher in IVIG non-responders than in IVIG responders. In the afebrile stage, there was no difference between IVIG responders and non-responders. The sRAGE levels increased progressively with the course of disease, but increased slowly in IVIG non-responders. In the afebrile and subacute stages of KD, sRAGE levels were lower in IVIG non-responders than in IVIG responders.

**Table 2:** Serum levels of resistin, S100A12 and soluble receptor for advanced glycation end products (sRAGE) in intravenous immunoglobulin (IVIG) responding or non-responding patients with Kawasaki disease

Characteristic		IVIG responders (*n *= 36)	IVIG non-responders (*n *= 4)	*P*
Boys/girls, number		21/15	1/3	0.000
Age, months		22.4 (17.2)	30.5 (12.8)	0.000
Patients with CALs, number		7	2	0.000
Resistin, 10^2 ^ng/ml	Acute stage	0.35 (0.24)	0.64 (0.30)	0.032
	Afebrile stage	0.21 (0.19) **	0.48 (0.35)	0.019
	Subacute stage	0.12 (0.05) **^▲^	0.28 (0.19)	0.000
S100A12, 10^2 ^ng/ml	Acute stage	5.05 (3.22)	7.92 (2.61)	0.044
	Afebrile stage	3.99 (3.49)	4.80 (4.26)	0.668
	Subacute stage	2.35 (2.26)** ^▲^	4.98 (4.75)	0.047
sRAGE, 10^2 ^pg/ml	Acute stage	3.07 (1.98)	2.37 (0.96)	0.493
	Afebrile stage	6.00 (2.78)**	3.51 (2.64)	0.046
	Subacute stage	7.19 (2.88)**	3.65 (3.27)	0.026

Results are mean (SD) unless stated otherwise. CALs, coronary artery lesions; **P < 0.01 compared with acute stage; ^▲^*P *< 0.05 compared with afebrile stage.

### Serum levels of resistin, S100A12, sRAGE in KD patients with and without CALs

**Figure 2:**
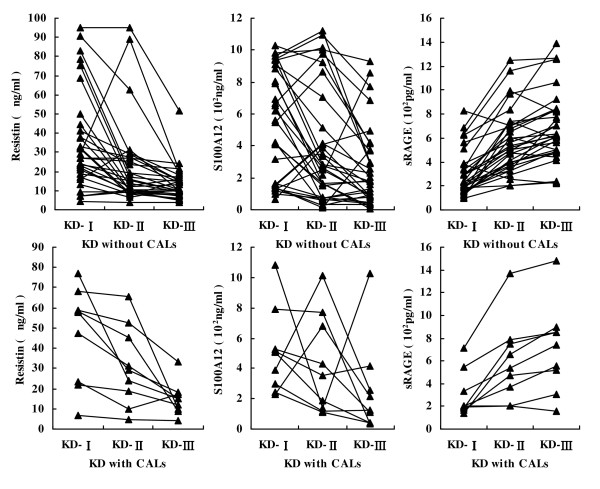
**Resistin, S100A12 and soluble receptor for advanced glycation end products (sRAGE) levels at different stages of KD in patients with and without coronary artery lesions (CALs)**. KD-I, acute stage; KD-II, afebrile stage; KD-III, subacute stage.

Serum levels of resistin, S100A12 and sRAGE in KD patients with and without CALs are shown in Table 3 and Figure 2. Nine KD patients (four boys and five girls) developed CALs. In all KD patients, resistin and S100A12 levels decreased progressively, and sRAGE levels increased progressively with the course of disease. Resistin, S100A12 and sRAGE levels did not differ significantly between KD patients with CALs and without CALs.

**Table 3:** Serum levels of resistin, S100A12 and soluble receptor for advanced glycation end products (sRAGE) in Kawasaki disease (KD) with or without coronary artery lesions (CALs)

Characteristic		KD with CALs (*n *= 9)	KD without CALs (*n *= 31)	*P*
Boys/girls, number		4/5	18/13	0.000
Age, months		27.1 (20.7)	22.1 (15.6)	0.001
Resistin, 10^2 ^ng/ml	Acute stage	0.46 (0.24)	0.36 (0.26)	0.268
	Afebrile stage	0.31 (0.20)	0.22 (0.22) *	0.266
	Subacute stage	0.15 (0.08)** ^▲^	0.13 (0.08) **	0.663
S100A12, 10^2 ^ng/ml	Acute stage	5.09 (2.78)	5.40 (3.42)	0.804
	Afebrile stage	4.22 (3.29)	4.02 (3.63)	0.883
	Subacute stage	2.52 (3.16)	2.65 (2.53)**	0.899
sRAGE, 10^2 ^pg/ml	Acute stage	2.97(1.99)	3.01 (1.92)	0.959
	Afebrile stage	5.93 (3.59)*	5.70 (2.64)**	0.833
	Subacute stage	7.06 (3.88)*	6.77 (2.86)**	0.802

Results are mean (SD) unless stated otherwise. *P < 0.05, **P < 0.01 compared with acute stage; ^▲^*P *< 0.05 compared with group afebrile stage.

### Correlation between different parameters in patients with KD

Table 4 shows the partial correlation coefficients of different parameters in patients with KD. Correlation was adjusted for gender and age. Significant correlations were found between resistin, S100A12, sRAGE and established inflammatory agents. Resistin levels were positively correlated with S100A12 levels. The sRAGE levels were not only negatively related with S100A12 levels, but also with resistin levels, the product of resistin and S100A12, the ratio of resistin to sRAGE, the ratio of S100A12 to sRAGE, WBCs, neutrophils and CRP. Resistin and S100A12 levels were positively correlated with the ratio of resistin to sRAGE, the ratio of S100A12 to sRAGE, WBCs, neutrophils and CRP.

**Table 4:** Partial correlation coefficients of different parameters in patients with Kawasaki disease

	S100A12	sRAGE	Resistin × S100A12	Resistin/sRAGE	S100A12/sRAGE	WBCs	N	CRP
Resistin	0.552***	-0.507***	0.889***	0.866***	0.696***	0.500***	0.695***	0.613***
S100A12		-0.252*	0.777***	0.376**	0.734***	0.339***	0.347***	0.321**
sRAGE			-0.374***	-0.575***	-0.547***	-0.533***	-0.614***	-0.566***
S100A12 × Resistin				0.701***	0.771***	0.385***	0.530***	0.448***
Resistin/sRAGE					0.782***	0.542***	0.734***	0.668***
S100A12/sRAGE						0.529***	0.609***	0.567***
WBCs							0.523***	0.449***
N								0.639***

sRAGE, soluble receptor for advanced glycation end products; WBCs, white blood cells; N, neutrophils; CRP, C-reactive protein. Control variables were age and gender. *Correlation significant at the 0.05 level (2-tailed); **correlation significant at the 0.01 level (2-tailed); ***correlation significant at the 0.001 level (2-tailed).

## Discussion

As expected, this study found that the KD patients in the acute stage had elevated resistin and S100A12 serum levels compared with healthy children. The same situation included the product of resistin and S100A12, the ratio of resistin to sRAGE and the ratio of S100A12 to sRAGE. The phenomena show that various types of inflammatory proteins are activated in KD [22,23]. However, sRAGE levels decreased in KD patients compared with healthy children. Serum levels of resistin and S100A12 in KD patients were peaked in the acute phase before IVIG, and returned to approximately normal levels in the afebrile and subacute stages, respectively. Resistin, S100A12, WBCs, neutrophils, and CRP tended towards a similar pattern of change, which was the opposite of the changes in expression of sRAGE, lymphocytes, monocytes and platelets. Resistin and S100A12 have similar significance to established inflammatory factors in KD patients. Although sRAGE could not be fully confirmed as a protective factor, it can still be a sensitive index response to attack by pro-inflammatory agents like S100A12 or resistin, in view of its negative correlation with them.

Resistin is known to be secreted by adipose tissue and by macrophages and monocytes [3,4], while S100A12 is normally secreted by neutrophils, with low expression on lymphocytes and monocytes [15]. Although serum levels of S100A12 were significantly higher than the levels of resistin in patients with KD, the change trends were coincident for both S100A12 and resistin in the different stages of KD. A markedly positive correlation between serum resistin and S100A12 levels in KD has been highlighted. Serum resistin and S100A12 levels have shown a negative correlation with sRAGE. Moreover, resistin expression is notably correlated with neutrophils, but the relationship between the sources of resistin and neutrophils still remains unclear. However, we believe that both of them are significant in the process of KD and it seems that resistin, S100A12, and sRAGE are three elements of a balance with positive and negative feedbacks, which could regulate the pathophysiology of KD.

IVIG was not effective for all the children with KD. Patients who did not respond had age and gender differences compared with responders. The response to IVIG depends on children's physiological functions and pathologic state. In the acute, afebrile and subacute stages of KD, resistin levels were higher in IVIG non-responders than in responders, as were S100A12 levels in the acute and subacute stages of KD. This suggests that the inflammatory reaction is more severe in IVIG non-responders than in responders. In the acute stage before IVIG treatment, sRAGE levels were not significantly different in non-responders compared with responders. In the afebrile and subacute stages of KD, sRAGE levels were lower in IVIG non-responders than in responders. This demonstrates that the protective factor against inflammation is lower in IVIG non-responders than in responders. It suggests that it is possible to apply ectogenesis sRAGE to alleviate the inflammatory reaction in IVIG non-responders.

The average serum levels of resistin, S100A12 and sRAGE were not significantly different in the KD patients with CALs compared to those without CALs. Serum levels of resistin, S100A12 and sRAGE are unlikely to predict the risk of CALs in KD patients. The results are consistent with a recent study [24].

To our knowledge, the present study is the first simultaneous evaluation of resistin, S100A12 and sRAGE in the acute, afebrile and subacute stages of KD. Also it is the first report of higher resistin levels in IVIG non-responders compared to IVIG responders in the acute, afebrile and subacute stages of KD; higher S100A12 levels in IVIG non-responders than in IVIG responders in the acute and subacute stages of KD; and lower sRAGE levels in IVIG non-responders than in IVIG responders in the afebrile and subacute stages of KD.

## Conclusions

In KD, sRAGE levels appear to reverse regulation with the pro-inflammatory factor resistin and S100A12 levels. The balance between the levels of resistin, S100A12 and sRAGE may represent a dynamic system with the interaction of positive and negative feedbacks. It suggests that the resistin, S100A12 and sRAGE are involved in the pathophysiology of KD.

## Abbreviations

ANOVA: analysis of variance; CAD: coronary artery disease; CAL: coronary artery lesion; CRP: C-reactive protein; CVD: cardiovascular disease; ELISA: enzyme-linked immunosorbent assay; ENRAGE: extracellular newly identified RAGE-binding protein; ESR: erythrocyte sedimentation rate; KD: Kawasaki disease; IVIG: intravenous immunoglobulin; LSD: least significant difference; RAGE: receptor for advanced glycation end products; sRAGE: soluble receptor for advanced glycation end products; WBC: white blood cell.

## Competing interests

The authors declare that they have no competing interests.

## Authors' contributions

YQ and QZ carried out the ELISA and statistical analysis, and drafted the manuscript. CX, WW and SF participated in the collection, analysis and interpretation of data. FG participated in the design, analysis and interpretation of data, drafting and revision of manuscript. All authors read and approved the final manuscript.
